# Practical application of the facial fat compartments and the line of ligaments concept in achieving the lifting effect in the lower face region in female patients

**DOI:** 10.1097/JW9.0000000000000080

**Published:** 2023-04-06

**Authors:** Lidia Majewska

**Affiliations:** a ESME Clinic & ul. Lwowska 1/U16, 30-548, Kraków, Poland

**Keywords:** facial fat compartments, lower face region, lifting effect, line of ligaments, soft tissue HA filler

**What is known about this subject in regard to women and their families?**

- Facial aging is a complex process that involves all the tissues of the face, including bones, muscles, ligaments, fat, and skin.
- The visible signs of facial aging result from soft tissue descent, facial volume loss, and alterations in skin texture and color.
- The midface is particularly prone to displaying signs of facial aging due to changes in the underlying bony platform and fat compartments.
- While the midface has traditionally been the focus of facial rejuvenation treatments, recent research has shown that addressing other areas of the face can lead to pan-facial effects.

**What is new from this article as messages for women and their families?**

- Novel treatment strategies have been developed that address the line of ligaments, facial fat compartments, and facial fascial layers to achieve the most natural and esthetic outcomes.
- The lateral temporal cheek fat compartment, in particular, has been found to be one of the most potent facial regions for inducing pan-facial effects. Injection therapy in this region has been shown to result in beneficial changes to the forehead, medial, and lateral midface.
- By addressing the lateral temporal cheek fat compartment, clinicians can achieve a more natural and youthful appearance in the entire face using moderate amounts of hyaluronic acid fillers.

## Dear Editors,

The rhytidectomy has always been the gold standard in treating facial sagging and loss of facial contour, however recent years brought better understanding of the facial anatomy and facial aging processes, especially those affecting fat pads and ligaments. Understanding the fact that face is not a static structure but a 3D dynamic one allows for applying new injection techniques to achieve natural results.

**Fig. 1.**
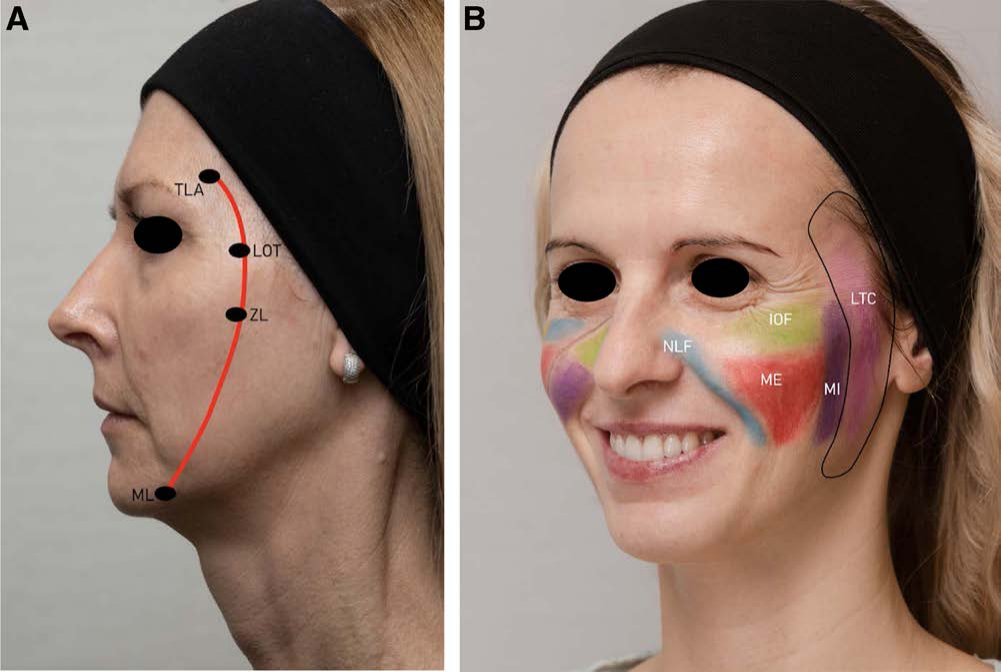
Anatomy of the face—with special focus on the line of ligaments and superficial fat compartments. (A) Concept of the line of ligaments (B). Visualization of the superficial facial fat compartments. Lateral temporal cheek fat compartment additionally marked in black. IOF, infraorbital fat compartment; LOT, lateral orbital thickening; LTC, lateral temporal cheek fat compartment; ME, medial fat compartment; MI, middle fat compartment; ML, mandibular ligament; NLF, nasolabial fat compartment; TLA, temporal ligamentous adhesion; ZL, zygomatic ligament.

The concept of subcutaneous facial fat compartments^1^ suggests that the face does not age as a composite mass. The shearing forces between neighboring compartments may be the reason behind soft tissues malposition. This knowledge, together with the concept of the line of ligaments (Fig. 1A) and the preconditioning effect of the lateral injection^2,3^ allows to gain an aesthetic lifting effect in the lower face area.

The technique reported here is based on the filling of the superficial lateral temporal cheek fat compartment (Fig. 1B). Specific targeting of this region with the moderate amount of hyaluronic acid (HA) can lift the position of soft tissues of the lower face and accentuate the jawline contour. Novelty of this technique is based on the fact that this region of the face is usually not a target area for the injection procedures using HA fillers. In recent years, however, there was a number of publications on non-surgical temporal lifting techniques proving their efficacy in the correction of facial shape.^2–5^ In author’s personal experience, combining temporal lifting with the presented here technique of the lateral non-surgical lifting is more beneficial toward improving facial contours.

To assess the aesthetic improvement of the treated area, the Global Aesthetic Improvement Scale (GAIS) was used. The GAIS is a scale where 0 = worse, 1 = no change, 2 = improved, 3 = much improved, and 4 = very much improved. The patient was also asked to report her level of personal satisfaction with the treatment (very satisfied, satisfied, not satisfied) and whether she would recommend the treatment to her friends (yes or no).

**Fig. 2.**
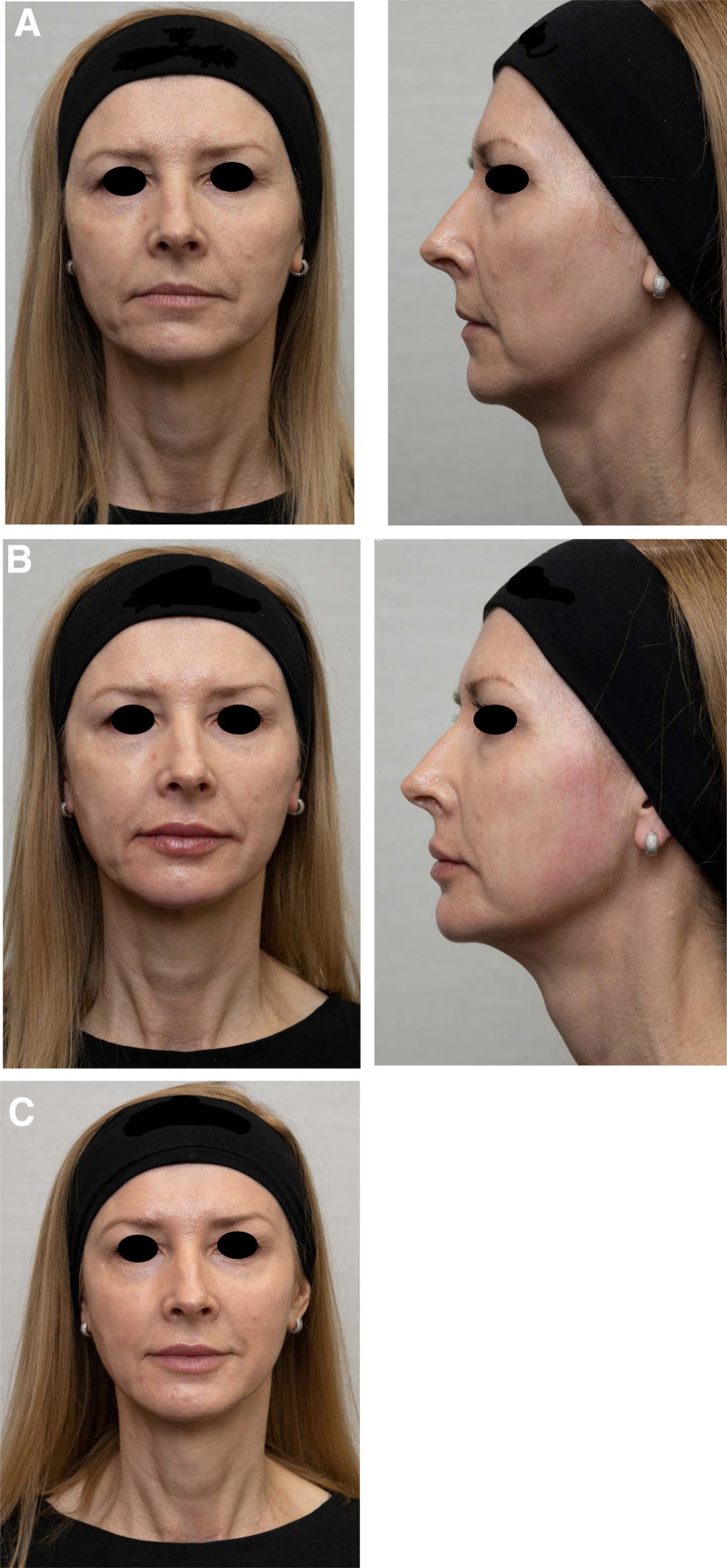
Images of the patient before the treatment (A), immediately after the treatment (B), and 2 wks post-treatment (C).

The patient presented here is a 56-year-old woman. She had no previous soft tissue filler treatments of the face, reported absence of previous thread treatment, and had no previous face or neck surgery. Medical history revealed an absence of allergies, contraindications, or chronic diseases that would exclude the application of soft tissue fillers in the face (Fig. 2).

After topical skin disinfection (Skinsept Pur; Ecolab Deutschland GmbH, Düsseldorf, Germany), it was punctured 1.5 cm to the Porion with a 23 G needle and a 25 G 50 mm blunt cannula (TSK, Japan) was inserted in the downward direction. The entire length of the cannula was advanced in the superficial subdermal plane until reaching the mandibula angle. Using the fanning technique, a total volume of 0.7 mL of the product (TEOSYAL RHA 4; Teoxane, Geneva, Switzerland) was injected in a triangular shape starting from the mandibular angle all the way to the anterior border of the superficial lateral temporal cheek fat compartment. The cannula was then withdrawn to the point where direction change was possible and it was redirected toward the temporal region and one bolus (0.5 mL) of the HA filler (TEOSYAL RHA 4) was injected in a region of the posterior temple. The post-procedure cream (Post Procedure; Teoxane) containing arnica extract and chlorhexidine was applied together with a slight massage of the treatment area.

The immediate effect of the treatment in terms of improving contour of the jaw, accentuation of the mandibular angle, and decreasing of the marionette lines was observed by the patient, the treating physician, and an independent observer. To further improve the effect and the appearance of the lower face, slight augmentation of the lips was performed using 1 mL of the HA filler (TEOSYAL RHA 2; Teoxane) with 0.4 mL of the product being placed along the contour of the lips with a 30 G 13 mm needle and 0.6 mL of the product placed subdermally in the vermillion using a 25 G 38 mm blunt cannula (TSK).

Immediately after the treatment, slight erythema over the injection site was observed. It diminished after 2 hours. The patient reported slight tension, swelling, and sensitivity in the treated area during 24 hours after injection. The patient was advised to apply cold packs over the treated area—every hour, for 10 minutes. All above reported problems (swelling, sensitivity) resolved without medical intervention within <48 hours. Patient satisfaction was additionally increased by the instant effect of the treatment and the ability to regain daily activities due to lack of post-treatment down time.

The GAIS was assessed 2 weeks after the treatment. The evaluation of GAIS relied on the assessment by the patient, by the treating physician and one independent observer. The patient rated her overall facial improvement as very much improved. The treating physician and independent observer rated the effect as much improved. Additionally, the patient reported her level of personal satisfaction with the treatment as “very satisfied” and stated that she would definitely recommend the treatment to her friends. The results of the retrospective image analysis (Fig.2) and GAIS assessment confirmed the positive outcome and the role of the temporal lateral cheek fat compartment in soft tissue filler injections for aesthetic facial treatments. The subdermal product placement in the described area had its effect in the lower face by visibly improving the contour of the jawline and the mandibular angle.^4,5^ The author’s experience shows that the lifting effect generated by traction of the skin toward auricular region while applying this technique allows for use of smaller amounts of HA fillers in the zygomatic region, decreases the visibility of the nasolabial folds and marionette lines. Key learning points are: proper patient assessment and qualification for the procedure. After excluding medical contraindications for using injectable fillers, it is important to assess site of the planned injections. Patients who would benefit the most are those with no excess fat in the lower face, those with resorption of the lateral temporal cheek fat compartment and skin laxity. Thin and lax skin is not a contraindication however in those patients, choice of HA filler would be very important to avoid irregularities. Preferred here are HA fillers having high lifting capacity but at the same time easily adapting to facial movements and facial dynamism. Product has to be placed superficially under the skin, using the fanning technique with a simultaneous skin lifting motion by the non-dominant hand. The described technique preserves facial identity and can be recommended to achieve a lifting effect in the lower face using moderate amounts of soft tissue HA fillers.

## Conflicts of interest

None.

## Funding

None.

## Study approval

This study (retrospective analysis) was conducted in accordance with regional laws (Poland) and good clinical practice. The patients provided written informed consent for the use of both their data and associated images prior to the treatment.
